# Looking for Pyroptosis-Modulating miRNAs as a Therapeutic Target for Improving Myocardium Survival

**DOI:** 10.1155/2015/254871

**Published:** 2015-09-27

**Authors:** Seahyoung Lee, Eunhyun Choi, Min-Ji Cha, Ki-Chul Hwang

**Affiliations:** ^1^Institute for Bio-Medical Convergence, College of Medicine, Catholic Kwandong University, Gangneung-si, Gangwon-do 210-701, Republic of Korea; ^2^Catholic Kwandong University International St. Mary's Hospital, Incheon Metropolitan City 404-834, Republic of Korea

## Abstract

Pyroptosis is the most recently identified type of regulated cell death with inflammatory response and has characteristics distinct from those of apoptosis or necrosis. Recently, independent studies have reported that small noncoding RNAs termed microRNAs (miRNAs) are involved in the regulation of pyroptosis. Nevertheless, only a handful of empirical data regarding miRNA-dependent regulation of pyroptosis is currently available. This review is aimed to provide a current update on the role of miRNAs in pyroptosis and to offer suggestions for future studies probing miRNAs as a linker connecting pyroptosis to various cardiovascular diseases (CVDs) and their potential as a therapeutic target for preventing excessive cell death of myocardium during CVDs.

## 1. Introduction

Heart disease has been the leading cause of death worldwide for many decades [1], and the loss of cells in the myocardium mutually affects the development of various heart diseases with functional demise of myocardium that can ultimately result in heart failure. In a dire situation such as ischemia, where nutrients and oxygen are deprived, individual cells are subjected to a live or die decision, and various cell death mechanisms can be activated as results [2, 3]. Among different cell death mechanisms, pyroptosis is the most recently recognized form of programmed cell death. Pyroptosis, first discovered by Cookson and Brennan in 2001, is characterized by cell lysis and inflammatory cytokine release [4]. Pyroptosis differs from apoptosis in that it does not show the typical membrane blebbing of apoptosis while inducing cell lysis, swelling, and pore formation that are not observed in apoptosis. Furthermore, the type of cysteine-dependent aspartate-specific proteases (caspases) that orchestrates disassembly of the cells in pyroptosis is caspase-1 as opposed to the caspase-3 of apoptosis [2]. In an evolutionary point of view, it is believed that pyroptosis initially developed as a host defense against microbial infections [5]. However, pyroptosis can be induced under noninfectious conditions. For example, typical signs of pyroptosis such as upregulation of caspase-1 and interleukin 1*β* (IL-1*β*) in myocardial infarction (MI) have been reported [6], suggesting that modulation of pyroptosis may be a viable therapeutic target for alleviating selective cardiovascular diseases such as MI.

During the last decade, a new class of small, noncoding RNAs termed microRNAs (miRNAs) has emerged as a key regulator of cellular process such as survival, differentiation, and death [7, 8]. Naturally, it is not too far-fetched of an assumption that the development of pyroptosis is also regulated by miRNAs to some extent, and we aim to summarize currently known pyroptosis-modulating miRNAs and others might be involved in pyroptosis based on its expressions in physiologic/pathologic hearts and the analysis of predicted targets. Through this mini review, we aim to provide a glimpse of how miRNA-dependent regulation of pyroptosis fits into a bigger picture of physiologic and pathologic regulation of heart cells by examining any possible connections of those miRNAs to other cardiovascular diseases (CVDs). Furthermore, we hope to offer constructive suggestions for conducting future studies investigating the role of miRNAs in pyroptosis and their potential as a therapeutic target for preventing undesired loss of the heart cells in CVDs.

## 2. MicroRNAs at a Glance

MicroRNAs are relatively short (approximately 21~23 nucleotides long), noncoding RNAs that bind to target mRNAs with complementary sequences for degradation and/or translation repression of the target mRNAs, thus acting as a posttranscriptional regulator of genes [9]. Initially, miRNA is transcribed as usually thousands of nucleotide long, primary transcript called pri-miRNA by RNA polymerase II in the nucleus. This pri-miRNA is subsequently processed by the ribonuclease III Drosha to produce approximately hundred nucleotides long premature miRNA (pre-miRNA) with hairpin-like structure. After being transferred to the cytosol by the nuclear export factor Exportin 5, pre-miRNA is further processed by ribonuclease III Dicer to produce a mature miRNA [10]. The double stranded mature miRNA has a short-life so it promptly unwound into two strands and more stable strand associates with argonaute (Ago) protein, which comprises a RNA-induced silencing complex (RISC) [11]. The RISC utilizes miRNA as a template to recognize the complementary sequence in the 3′ untranslated region (UTR) of target mRNA [12]. As a result, gene silencing is achieved via either hindered mRNA translation or mRNA degradation [13]. Since their first discovery in 1993 [14], miRNAs have been implicated in various diseases including CVDs [15–17]. Pertaining to cardiomyocyte death, the importance of miRNA-mediated regulations in cardiac apoptosis [18, 19] and autophagy [20, 21] has been reported.

## 3. Pyroptosis

Meaning “fire + falling” in Greek, pyroptosis is a proinflammatory cellular suicide program and part of the host defense system responding to pathogens. Initial step of pyroptosis is the formation of caspase-1 binding protein complexes called inflammasome, and this is triggered by the cytosolic receptor-mediated recognition of bacterial and/or viral pathogens or host danger signals such as pathogen-associated molecular patterns (PAMPs) and/or damage-associated molecular patterns (DAMPs) [2, 22].

### 3.1. Inflammasome

Inflammasome generally consisted of a cytosolic pattern-recognition receptor (PRR), caspase-1, and an adaptor protein that connects caspase-1 to the cytosolic PRR [22].

#### 3.1.1. Cytosolic PRRs

The cytosolic PRRs function as cytosolic molecular sensors and they are either NOD-like receptor (NLR) family proteins or pyrin and HIN domain-containing (PYHIN) proteins [23]. In humans, the NLRs family consists of 22 members and has 3 distinctive subfamilies: the NODs (NOD1-2, NOD3/NLRC3, NOD4/NLRC5, NOD5/NLRX1, and CIITA), the NLRPs (NLRP1-14, also known as NALPs), and IPAF (NLRC4 and NAIP) [24]. Each NLR contains three distinct domains, namely, an N-terminal effector domain, a central nucleotide binding and oligomerization (NACHT) domain, and a C-terminal leucine-rich repeat (LRR) domain. The N-terminal effector domain facilitates signal transduction and four different types have been identified: acidic transactivation domain, caspase activation and recruitment domain (CARD), pyrin domain (PYD), and baculoviral inhibitor of apoptosis protein (IAP) repeat domain. The NACHT domain facilitates signaling complex activation via ATP-dependent oligomerization, and the C-terminal LRR domain is responsible for ligand sensing and autoinhibition [25].

#### 3.1.2. Caspase-1 and Adaptor Protein

Formerly known as interleukin 1*β* converting enzyme (ICE), caspase-1 was the first caspase identified in mammals [26]. In humans, caspase-1, caspase-4, caspase-5, and caspase-12 comprise inflammatory caspases [27] as opposed to apoptotic caspases such as caspase-3, and, as a member the inflammatory caspases, caspase-1 does not contribute to apoptosis. In relation to CVDs, caspase-1-mediated cardiomyocyte death has been reported [28]. Like caspase-9 which is activated via formation of apoptosome with apoptotic protease-activating factor-1 (Apaf-1) during apoptosis [29], caspase-1 is activated through interaction with inflammasome during the process of pyroptosis. At resting state, NLR monomers stay in inactive conformation. Upon activation, NLR monomers oligomerize via homotypic interaction of NACHT domains and bind to the adapter protein called apoptosis-associated speck-like protein containing a CARD (ASC/PYCARD) through PYD-PYD interaction [30]. Subsequently, these adaptor proteins recruit procaspase-1 via homotypic CARD interaction and cleave to activate the recruited procaspase-1 by induced proximity mechanism [31, 32]. The major mechanism of activated caspase-1-mediated pyroptosis is the formation of ion-permeable pores in the plasma membrane which creates osmotic pressure driving water influx, subsequent cell swelling, and eventual cell lysis with the release of intracellular content [33]. These released cytoplasmic contents become DAMPs and trigger further pyroptotic cascade [34]. Additionally, the activated caspase-1 mediates maturation of IL-1*β* and -18 inducing inflammatory responses [35]. Although this caspase-1-mediated inflammatory response was proven not to be indispensable for cell death [36], the significant role of both IL-1*β* and -18 in the development and progression of CVDs has been reported [37–39].

## 4. Approaches to Finding Candidate Pyroptosis-Modulating miRNAs

Currently, only few miRNAs have been identified as meaningful mediators of pyroptosis. However, this insufficient empirical data on the miRNA-dependent regulation of pyroptosis does not necessarily indicate that the role of miRNAs in the development and progression of pyroptosis is insignificant. Rather, it is probably due to the fact that this field of miRNA-mediated regulation of pyroptosis is in its infancy and the number of studies on this subject is anticipated to increase in upcoming years. Thus, in this section, we try to offer some feasible research strategies for future studies focusing on how to systematically downsize the number of candidate miRNAs suspected to be involved in the development and progression of pyroptosis. Additionally, currently available information of the miRNA-dependent regulation of pyroptosis will be discussed where appropriate.

### 4.1. Scenario 1: miRNAs Directly Targeting Key Mediators of Pyroptosis

This is the most intuitive and straightforward case, and the most reasonable approach will be finding miRNAs directly targeting key protein-based mediators of pyroptosis such as pattern recognition receptors (NOD-like receptor (NLR) family of proteins), adaptor proteins, and caspase-1 based on miRNA databases such as, but not limited to, TargetScan [40] and miRWalk [41]. In this case, the first task to be done is to list up possible targets, along with a list of miRNAs (referred to as predicted miRNAs) predicted to target each of the specific proteins of interest (i.e., individual NLR proteins). Next, miRNAs that are dysregulated (referred to as dysregulated miRNAs) in CVDs have to be summarized from individual research articles or review articles providing miRNAs profile in CVDs. Of note, in this particular scenario, “dysregulated” especially means “decreased” because miRNAs are generally a negative regulator of target genes. For example, recent studies have reported that miR-223 directly targets NLRP3 acting as a negative regulator of inflammasome formation [42, 43]. Those were two of the few studies that addressed the evidence of miRNA-dependent regulation of pyroptosis. In this particular case, if miR-223 were to be a link between pyroptosis and CVDs, the expression level of miR-223 would be decreased in CVDs of interests so that the expression of NLRP3 can be increased suppressing inflammasome formation. Nevertheless, we could only identify that one study reported decrease of miR-223 in CVDs [44], and further association between the downregulation of miR-223 and CVDs could not be found. Now, back to the bioinformatic screening process, by crosschecking the dysregulated miRNAs and the predicted miRNAs, one can logically conduct a rough screening of miRNAs for possible involvement in both pyroptosis and CVDs, as exemplified in Table 1.

For example, miR-1 has been reported to be downregulated in various CVDs including myocardial infarction (MI) [45–47], and it is predicted to target multiple NLR proteins (NOD1, NLRP14, and NAIP). Since it has been also documented that acute MI increased caspase-1 activity and formation of inflammasome [48], decreased miR-1 during MI might be an unappreciated propyroptosis mechanism by which the synthesis of inflammasome components is enhanced contributing to the development of pyroptosis. Nevertheless, such deductive reasoning should be empirically tested and validated. In such cases, exogenous delivery of corresponding miRNA mimics or finding ways to increase and/or maintain the endogenous level (i.e., small-molecules increasing specific miRNAs) would be appropriate.

### 4.2. Scenario 2: miRNAs Indirectly Affect Pyroptosis via Secondary Mediators

In this case, the premise is that miRNAs target regulators of pyroptosis (as opposed to components of inflammasome). This case can be further divided into two possible scenarios: miRNAs target negative regulators or miRNAs target positive regulators. Depending on the type of regulators, the expression patterns of miRNAs in CVDs to look for can be either downregulation or upregulation (Table 2).

Since the same strategy used for the scenario 1 can be applied for the case of miRNAs targeting positive regulators, only the case of miRNAs targeting negative regulators of pyroptosis will be covered in this section. A good example would be the NLRP3 inflammasome. The NLRP3 inflammasome has been recently reported to be involved in ischemia-reperfusion- (I/R-) induced myocardial injury [6], and number of negative regulators have been identified [49]. As laid out above, miRNAs targeting negative regulators should be increased in CVDs to facilitate enhanced pyroptosis. Selected examples of negative regulators and miRNAs targeting those regulators are summarized in Table 3.

#### 4.2.1. Autophagy-Mediated Suppression of Inflammasome Activation

Meaning “self-eating” in Greek, autophagy is a highly conserved process by which long-lived proteins and/or organelles are delivered to the lysosome and degraded within [50]. Negative regulation of pyroptosis by autophagy was demonstrated by the previous study reporting that knockout of Atg16L1 (autophagy-related-gene 16-like 1), one of the key factors of autophagy [51], significantly enhanced inflammasome activation and caspase-1 activation [52]. Additional key components of autophagy, namely, LC3B and beclin1 [53], also have been reported to function as negative regulators of pyroptosis by inhibiting activation of inflammasomes [54]. These data indicate that if miRNAs targeting those proautophagic proteins are upregulated during certain CVDs, those miRNAs can negate the negative regulation of pyroptosis by autophagic process resulting in enhanced pyroptosis.

#### 4.2.2. Transcription Factors Negatively Regulating Inflammasome Activation

Examples of transcription factors acting as a negative regulator of pyroptosis are signal transducers and activators of transcription 1 (STAT1) and forkhead box O3 (FOXO3a). Activated as a downstream signal of type I interferon (IFN) [55], STAT1 repressed the activity of NLRP1 and 3 inflammasomes subsequently hindering pyroptosis [56]. Consequently, miRNAs targeting STAT1 can act as a positive inducer of pyroptosis, and if such miRNAs are upregulated in CVDs, their potential as a linker between CVDs and pyroptosis will be worth examining. Another transcription factor that has been reported to be involved in negative regulation of pyroptosis is FOXO3a [57]. In that particular study, miR-30d increased downregulating one of its targets, FOXO3a, under a high-glucose condition. As a result, the apoptosis repressor with caspase recruitment domain (ARC) that is under the transcriptional regulation of FOXO3a [58] was decreased so that caspase-1 was upregulated and pyroptosis was induced. This was the last study providing the evidence of miRNA-dependent regulation of pyroptosis and is an excellent empirical example of scenario 2 where miRNAs (i.e., miR-30d) increased in CVDs (i.e., diabetic cardiomyopathy) and such increase caused downregulation of negative regulator of pyroptosis (i.e., ARC transcribed by FOXO3a).

#### 4.2.3. Nitric Oxide-Mediated Suppression of Inflammasome Formation

Nitric oxide (NO) has been reported to negatively regulate NLRP3 inflammasome-mediated caspase-1 activation [67]. Such results indicated that inducible nitric oxide synthase (iNOS), which is responsible for the production of NO [68], also can be a negative regulator of LNRP3 inflammasome. In fact, the significance of iNOS has been demonstrated in a study where IFN-*β* inhibited NLRP3 inflammasome [69]. Furthermore, since NO itself is not a protein that can be subjected to miRNA-dependent targeting, finding miRNAs that are increased in CVDs, as well as targeting iNOS, would be the next logical strategy for designing studies to elucidate the role of miRNAs in myocardial pyroptosis.

## 5. Concluding Remarks

Over the last few decades, tremendous interest has been placed on the regulatory nature of miRNAs under various pathologic conditions including CVDs. Considering the complexity of cell signaling in general, it is reasonable to assume that miRNAs are involved in virtually all biological processes including demise of cells. In this mini review, we tried to provide currently available evidences of miRNA-dependent regulation of pyroptosis. However, due to the limited number of studies, it was not sufficient to draw any significant insights on the role of miRNAs in pyroptosis, and it remains as one of the limitations of this review.

In addition to providing the evidences of miRNA-dependent modulation of pyroptosis, we also tried to offer some suggestions for future studies focusing on what to look for in search of the miRNAs modulating pyroptosis in CVDs. The aim of this review was to encourage scientific interest in examining the need-to-be-further-validated framework of CVD-miRNA-pyroptosis. We hope that, through future studies with well-thought-out strategies, our understanding of the subject of miRNA-dependent regulation of pyroptosis in CVDs is enhanced, and it contributes to the development of an optimized miRNA-mediated therapeutic strategy for targeting cell death of myocardium.

## Figures and Tables

**Table 1:** Examples of miRNA downregulated in CVDs with potential connection on pyroptosis.

Human inflammasome component (NCBI RefSeq)	miRNAs predicted by ^*^TargetScan and reported to be downregulated in CVDs	Related CVDs [reference]
NLR proteins (NODs)		
NOD1 (NM_006092)	miR-1	DCM, AS [45], MI [46], LVH [47]
	miR-133	MI [46], LVH [47]
	miR-208	MI [46]
	miR-499	AS [45], MI [46]
NOD2 (NM_022162)	miR-30e-5p	DCM, AS [45]
	miR-208	MI [46]
	miR-499	AS [45], MI [46]
NOD3/NLRC3 (NM_178844)	miR-10	AS [45]
	miR-24	MI [59, 60]
NOD4/NLRC5 (NM_032206)	miR-21	MI [61]
NOD5/NLRX1 (NM_024618)	miR-24	MI [59, 60]
	miR-133	MI [46], LVH [47]
CIITA (NM_000246)	miR-10	AS [45]
	miR-24	MI [59, 60]
NLR proteins (NOD-like receptor family pyrin domain containing, LNRPs)		
NLRP1 (NM_014922)	miR-10	AS [45]
NLRP2 (NM_017852)	No miRNA meets the screening criteria	
NLRP3 (NM_004895)	miR-17-5p	DCM [45]
	miR-30e-5p	DCM, AS [45]
NLRP4 (NM_134444)	No miRNA meets the screening criteria	
NLRP5 (NM_153447)	No miRNA meets the screening criteria	
NLRP6	Prediction data not available	
NLRP7 (NM_139176)	miR-150	MI [62]
NLRP8 (NM_176811)	miR-155	MI [62], LVH [63]
NLRP9 (NM_176820)	miR-19	DCM, AS [45]
NLRP10	Prediction data not available	
NLRP11 (NM_145007)	No miRNA meets the screening criteria	
NLRP12 (NM_033297)	No miRNA meets the screening criteria	
NLRP13	Prediction data not available	
NLRP14 (NM_176822)	miR-1	DCM, AS [45], MI [46], LVH [47]
NLR proteins (ice protease-activating factor (IPAF))		
NLRC4 (NM_021209)	No miRNA meets the screening criteria	
NAIP (NM_004536)	miR-1	DCM, AS [45], MI [46], LVH [47]
	miR-24	MI [59, 60]
	miR-150	MI [62]
Caspase		
Caspase-1 (NM_001223)	No miRNA meets the screening criteria	
Adaptor protein		
ASC/PYCARD (NM_013258)	No miRNA meets the screening criteria	

^*^In TargetScan search, only the “miRNA families broadly conserved among vertebrates” for each of the target proteins were used to generate this table. DCM: dilated cardiomyopathy, AS: aortic stenosis, MI: myocardial infarction, and LVH: left ventricular hypertrophy.

**Table 2:** Screening criteria of miRNAs, dependent on pyroptosis regulator type.

Type of regulators	Corresponding miRNA expressions in CVDs
Positive regulators (promote pyroptosis)	Decreased, abundant positive regulators expected
Negative regulators (inhibit pyroptosis)	Increased, depletion of negative regulators expected

**Table 3:** Selected examples of miRNAs targeting human negative regulators of pyroptosis and reported to be increased in CVDs.

Negative regulators of pyroptosis (NCBI Refseq, reference)	miRNAs predicted by ^*^TargetScan and reported to be upregulated in CVDs	Related CVDs [reference]
Autophagy		
ATG16L1 (autophagy-related gene 16 like-1) (NM_017974, [52])	Let-7	DCM, ICM, AS [45]
	miR-125	DCM [45, 64], AS [45], LVH [65]
	miR-181	DCM [64], AS [45]
	miR-214	DCM [45, 64], ICM, AS [45], LVH [65]
MAP1LC3B (microtubule-associated proteins 1A/1B light chain 3B) (NM_022818, [54])	miR-30e-5p	DCM, AS [45]
	miR-145	AS [45]
	miR-214	DCM, ICM, AS [45], LVH [65, 66]
	miR-221	LVH [65]
	miR-222	DCM, ICM [45], LVH [65]
BECN1 (Beclin1) (NM_003766, [54])	miR-30e-5p	DCM, AS [45]
	miR-199	DCM, ICM [45], LVH [65, 66]
Transcription factors		
STAT1 (NM_007315, [56])	miR-140	DCM, ICM, AS [45], LVH [65]
	miR-21	LVH [65, 66]
	miR-23	DCM, ICM, AS [45], LVH [65, 66]
	miR-24	ICM, AS [45]
** FOXO3**	miR-27	LVH [65, 66]
** **(NM_001455)	**miR-30d**	**Diabetic cardiomyopathy [57]**
** ** ^*^ **experimentally proven to**	miR-99	DCM, AS [45]
** be regulated by miR-30d** ** [57]**	miR-103	DCM, ICM, AS [45], LVH [65]
	miR-125	DCM, AS [45], LVH [65, 66]
	miR-217	LVH [66]
	miR-221/222	LVH [65]
NO synthase		
NOS2 (inducible nitric oxide synthase)	miR-15	DCM, AS [45]
(NM_000625, [67])	miR-214	DCM [64], LVH [65, 66]

^*^In TargetScan search, only the “miRNA families broadly conserved among vertebrates” for each of the target proteins were used to generate this table. DCM: dilated cardiomyopathy, AS: aortic stenosis, MI: myocardial infarction, and LVH: left ventricular hypertrophy.
